# In Situ Observation of Modulated Light Emission of Fiber Fuse Synchronized with Void Train over Hetero-Core Splice Point

**DOI:** 10.1371/journal.pone.0003276

**Published:** 2008-09-25

**Authors:** Shin-ichi Todoroki

**Affiliations:** Advanced Materials Laboratory, National Institute for Materials Science, Tsukuba, Ibaraki, Japan; Australian National University, Australia

## Abstract

**Background:**

Fiber fuse is a process of optical fiber destruction under the action of laser radiation, found 20 years ago. Once initiated, opical discharge runs along the fiber core region to the light source and leaves periodic voids whose shape looks like a bullet pointing the direction of laser beam. The relation between damage pattern and propagation mode of optical discharge is still unclear even after the first in situ observation three years ago.

**Methodology/Principal Findings:**

Fiber fuse propagation over hetero-core splice point (Corning SMF-28e and HI 1060) was observed in situ. Sequential photographs obtained at intervals of 2.78 µs recorded a periodic emission at the tail of an optical discharge pumped by 1070 nm and 9 W light. The signal stopped when the discharge ran over the splice point. The corresponding damage pattern left in the fiber core region included a segment free of periodicity.

**Conclusions:**

The spatial modulation pattern of the light emission agreed with the void train formed over the hetero-core splice point. Some segments included a bullet-shaped void pointing in the opposite direction to the laser beam propagation although the sequential photographs did not reveal any directional change in the optical discharge propagation.

## Introduction

**Figure 1:**
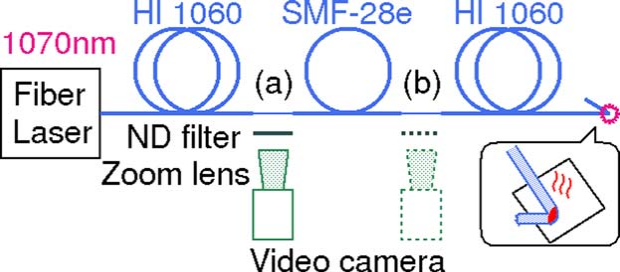
Experimental setup for observing fiber fuse propagation over hetero-core splice.

The fiber fuse effect was first reported in the late 1980's [1], [2]. It is initiated by the local heating of an optical fiber, which delivers a few watts of light, and generates an optical discharge running along the fiber to the light source at a speed of about 1 m/s (see the movie in the online version of [3] (Fig. 1) that shows a macroscopic view of fiber fuse propagation). This results in the catastrophic destruction of the core region. Thus, it has posed a real threat to every application where high power light is delivered through optical waveguides. Text S1 is a full list of related 70 papers.

The behavior of the rapidly moving optical discharge is the key to recognizing the destruction mode. Thus, ultra-high speed videography at nearly half a million frames per second is the most suitable tool for observing this behavior. With the aid of this technique, I investigated a collection of micrographs showing the optical discharge and the voids that remained after quenching to determining the periodic void formation mechanism in a single mode silica glass fiber pumped at 1480 nm [3], [4], [5].

In this previous work, the videography only showed that the optical discharge moved at a constant speed under a time resolution of 4 µs. In this paper, a state-of-the-art camera is used to observe the oscillating light emission of an optical discharge, which is found to be related to an irregular void track that remains over hetero-core splice point. In addition, a bullet-like void pointing in the opposite direction to the rest of voids was accidentally observed near the splice point although the propagation direction of the optical discharge had not changed. This information is useful for analyzing fiber fuse accidents involving high-power fiber lasers.

Japanese translation of this paper is available in Text S2.

## Methods

Figure **1** shows the experimental setup for observing fiber fuse propagation over a hetero-core splice. One end of a commercial single-mode silica glass optical fiber (SMF-28e, Corning, see Table 1) was connected to a Yb fiber laser (PLM-10-1070, IPG Laser, 1.07 µm, 9 W) with an HI 1060, Corning, output fiber (see Table 1). The other end of the fiber was spliced to another HI 1060 fiber. These two points are referred to as (a) and (b), respectively, in this paper. Hetero-core splicing was performed with a fusion splicer (S183, Furukawa Electric) containing a program for this pair.

**Table 1:** Specifications of the fibers used in this study.

	HI 1060	SMF-28e
Cutoff wavelength	920±50 nm	>1280 nm
Mode-field diameter	6.2±0.3 µm @ 1060 nm	9.2±0.4 µm @ 1310 nm
Core diameter	n. a.	8.2 µm
Cladding diameter	125 µm	125 µm

Source: Product information sheets at http://www.corning.com/opticalfiber/

A fiber fuse was initiated at the end of a fiber attached to a metallic plate. The propagation over one of the two splice points was observed through an ultra-high speed CCD camera (FASTCAM SA1.1, monochrome version, Photron Ltd., sensitivity range: 380–790 nm) with an appropriate zoom lens. Pictures with a resolution of 256×32 with a 1024-step gradation were taken every 2.78 µs (360,000 frames per second) with an exposure time of 0.37 µs through a neutral density (ND) filter (x8). The damaged sites were examined with an optical microscope.

## Results

**Figure 2:**
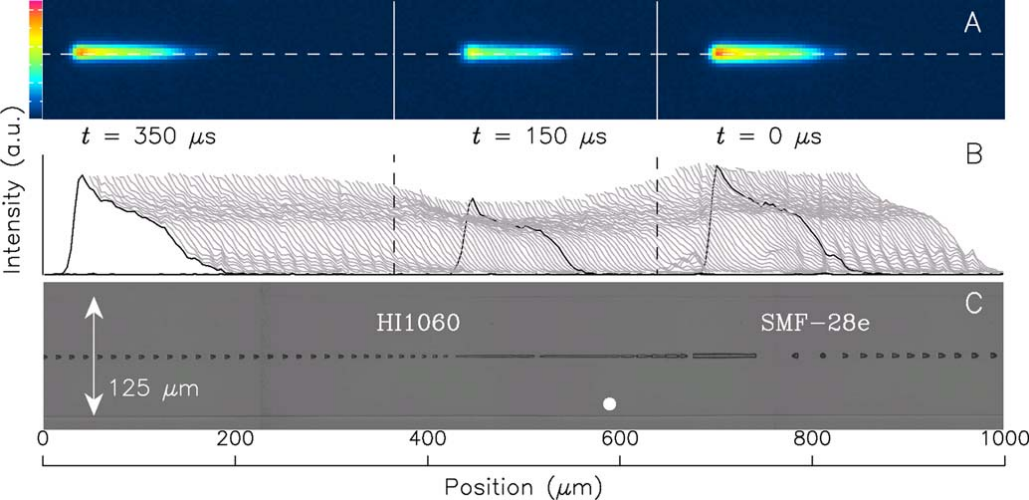
Visible light emission of a fiber fuse and generated voids near the splice point (a). A: Photographs of the visible light emission of a fiber fuse pumped by 1070 nm 9W light (original gray-scale image is converted to color-scale image), B: their intensity profiles along the dashed lines in these photographs taken every 2.78 µsec, and C: optical micrographs of the damage pattern generated at corresponding segments immersed in matching oil. The splice point is located near the solid white circle. The cladding diameter is 125 µm. The two dashed vertical lines represent the position at which the optical discharge changed its speed. See also Movie S1.

**Figure 3:**
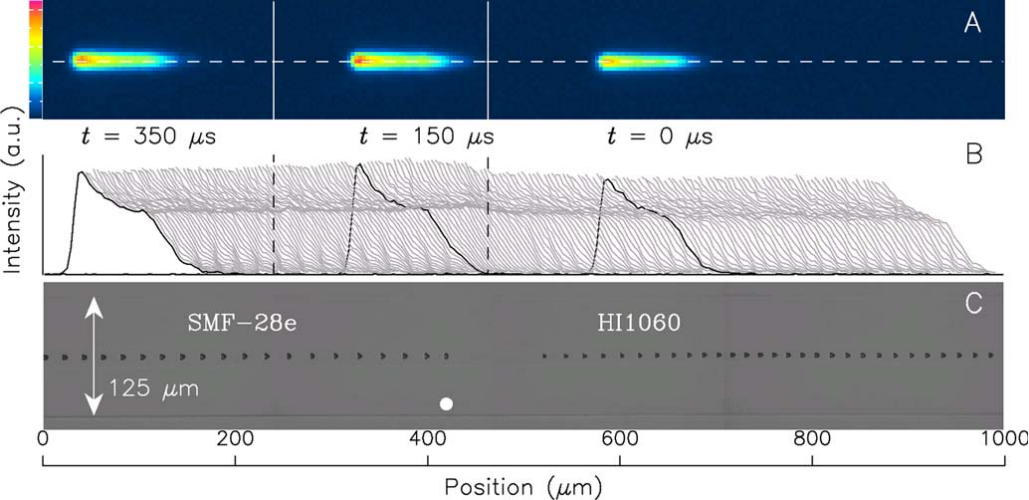
Visible light emission of a fiber fuse and generated voids near the splice point (b). See also Movie S2.

Ten examples of fiber-fuse propagation were recorded and they all showed a similar tendency. Typical recordings are shown in Fig. **2** and **3**. The top row of the figures show visible light being emitted from the optical discharge running over one of the splice points. Since the fiber acted as a cylindrical lens, these images were expanded in the vertical direction. The middle row (B) shows a time-varying intensity profile of the emission along the dashed line shown in the top row.

The bottom photograph (C) shows the damage train that remained after the propagation. Each splice point is estimated to be near the solid white circle. The void train shows that the propagation mode of the optical discharge is modulated at around the splice point. Accordingly, its velocity changed twice, namely before and after it reached the splice point. The inflection points of its velocity were calculated by analyzing the video image and are indicated as two dashed vertical lines in the middle row (B), which represent the peak of the intensity profile at the moment of speed change.

The averaged speed, *v*, in each segment is listed in Table 2. The speed in HI 1060 on the downstream side is smaller than that of the upstream side because the pumping energy is reduced through two hetero-core splice points with an inevitable insertion loss.

**Table 2:** Average speed of optical discharge, *v*, and average time of one void formation, τ, around the spliced points shown in Fig. **2** and **3**.

	HI 1060	(a)	SMF-28e	...	SMF-28e	(b)	HI 1060
*v* (m/s)	2.08	1.69	1.42		1.40	1.51	1.88
τ (µs)	6.6	-	12.1		16.7	-	8.4

At the tail of the intensity profile shown in Fig. **2**B and **3**B, a small discrete peak appeared periodically and moved to rearward with decreasing height unless the discharge stayed near the splice point. This sub-peak oscillation was recorded in my previous work [3] but was not analyzed precisely owing to the poor resolution (256-step gradation taken every 4 µs) of the video camera. The oscillation cycle of this peak coincides with the period of one void formation, τ, listed in Table 2, which is calculated from the average speed and void interval.

**Figure 4:**
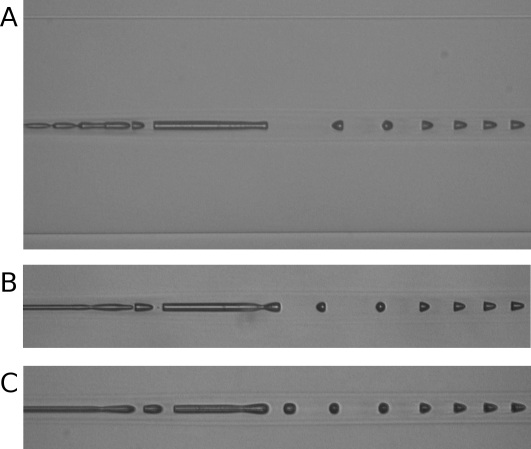
Optical micrographs showing fiber fuse damage generated near the splice point (a). The pump laser operates at 1070 nm and 9.0 W. The top is an expanded image of Fig. 2C.

Sometimes a single reverse bullet appeared just before the discharge running into the splice point (a) (see Fig. **2**C). Its expanded image is shown in Fig. **4**A together with other micrographs of the samples obtained under the same condition.

## Discussion

### Change in void pattern over splice point

Since the pump light (1070 nm) propagates in a multimode in SMF-28e, its energy density is lower than that in HI 1060. Thus, the propagation speed of the optical discharge is slower in SMF-28e than in HI 1060 as shown in Table 2. The reason for its speed changing twice, namely before and after passing over the splice point, is that a discharge with a length more than 100 µm running over the border remains in both HI 1060 and SMF-28e, i.e., in a transitional state.

In this transitional segment (shown between two vertical dashed lines in Fig. **2** and **3**), the splice point (shown as a solid white circle in these figures) is located on the downstream side of the pump laser. This is because the intensity profile of the fiber fuse emission along the fiber length is unsymmetrical.

Outside the transitional segment, the optical discharge generates a periodic void train. On passing through the splice point (b), the optical discharge reduces the void formation frequency (see Table 2). However, the interval of the periodic voids varies discontinuously around the border to form a void-free segment.

Considering the energy balance of fiber fuse propagation, the input is the pump laser energy and the outputs are the heating of materials, the emission of light and heat, the movement of the optical discharge and the creation of the void surface. Thus, in the transitional segment, these balance is modified to establish another equilibrium state. During the passage across the border (b), the optical discharge suspends void formation and slightly enhances the light emission. A similar phenomenon is observed in fiber fuse self-termination [5].

On the other hand, the frequency of periodic void formation increases after the optical discharge passed through the border (a). Then, the discharge in the transitional segment enhances the surface formation to construct a row of long voids with a reduction in light emission.

In both transitional segments, the optical discharge suspends the periodic oscillation of the sub-peak at its tail. This is clearly shown in the superimposed intensity profile patterns shown in Fig. **2**B and **3**B. The peak oscillation is recorded as a striped pattern, which is absent from these transitional segments. The stripe interval agrees with that of the periodic void train shown in Fig. **2**C and **3**C. In fact, the video images contain no information about the splice point. However, these striped patterns can be accurately superimposed on the void photograph.

Consequently, it is obvious that the sub-peak oscillation is related to the periodic void formation.

### Direction of bullet-shape in the damage train

It is well known that the bullet-like voids formed in ordinary fiber point in the pump light propagation direction. The formation mechanism of this shape is explained by the combined effect of the internal pressure of the optical discharge and the temperature gradient along the fiber [4]. Once a void is pinched off from the tail of the optical discharge, the cool side surface solidifies first leaving a spherical shape and the other hot side is pressed to form a plane.

However, Bufetov et al. reported that voids with a reversed direction are formed when there is a light intensity modulation along a fiber core introduced by an interference between LP and LP modes [6]. In this study, such a void sometimes appeared near the splice point (a) as shown in Fig. **4**. Since the video showed the discharge propagates without any change in direction, its formation mechanism is different from that described above. In fact, the striped pattern in Fig. **2**B for this reversed void is different from that for regular periodic voids. Thus, this phenomenon also seems to be caused by the light intensity modulation of hetero-core splicing.

## Supporting Information

Text S1 List of papers on fiber fuse.
(0.07 MB PDF)

Text S2 Japanese translation of this paper.
(1.11 MB PDF)

Movie S1 Movie version of Figure 2.
(0.36 MB MPG)

Movie S2 Movie version of Figure 3.
(0.40 MB MPG)
