# Supplementary material for: In Situ Observation of Modulated Light Emission of Fiber Fuse Synchronized with Void Train over Hetero-Core Splice Point
Source: PLoS One. 2008 Sep 25;3(9):e3276. doi: 10.1371/journal.pone.0003276 (PMC2542984; doi:10.1371/journal.pone.0003276)
Supplement: Text S1 — List of papers on fiber fuse. (0.07 MB PDF) [file pone.0003276.s001.pdf]

1 Fiber Fuse (2005-)

[1] N. Akhmediev, P. St. J. Russell, M. Taki and J. M. Soto-Crespo: "Heat dissipative solitons in optical fibers", Physics Letters A, **372**, 9, pp. 1531–1534 (2008).

[2] I. A. Bufetov, A. A. Frolov, A. V. Shubin, M. E. Likhachev, C. V. Lavrishchev and E. M. Dianov: "Fiber fuse effect: New results on the fiber damage structure", Proceedings of the 33rd European Conference on Optical Communication, Vol. 1, Berlin, Germany, IEE's Photonics Professional Network, pp. 79–80 (2007). (Mon 1.5.2). Homepage

[3] I. A. Bufetov, A. A. Frolov, A. V. Shubin, M. E. Likhachev, C. V. Lavrischev and E. M. Dianov: "Fiber fuse effect under conditions of interference of two modes", ICONO/LAT 2007 Technical Digest on CD-ROM, Minsk, Berarusi (2007). (L01/IV-2). Homepage

[4] S. I. Yakovlenko: "Physical processes upon the optical discharge propagation in optical fiber", Laser Physics, **16**, 9, pp. 1273–1290 (2006). Homepage

[5] E. D. Bumarin and S. I. Yakovlenko: "Temperature distribution in the bright spot of the optical discharge in an optical fiber", Laser Physics, **16**, 8, pp. 1235–1241 (2006). Homepage

[6] M. M. Lee, J. M. Roth, T. G. Ulmer and C. V. Cryan: "The fiber fuse phenomenon in polarization-maintaining fibers at 1.55μm", Proc. of the Conference on Lasers and Electro-Optics (CLEO) (2006). (JWB66).

[7] E. M. Dianov, V. E. Fortov, I. A. Bufetov, V. P. Efremov, A. A. Frolov, M. Y. Schelev and V. I. Lozovoi: "Detonation-like mode of the destruction of optical fibers under intense laser radiation", J. Exp. Theo. Phys. Lett., **83**, 2, pp. 75–78 (2006).
doi: 10.1134/S002136400602007X Homepage

[8] E. M. Dianov, V. E. Fortov, I. A. Bufetov, V. P. Efremov, A. E. Rakitin, M. A. Melkumov, M. I. Kulish and A. A. Frolov: "High-speed photography, spectra, and temperature of optical discharge in silica-based fibers", IEEE Photon. Technol. Lett., **18**, 6, pp. 752–754 (2006).
doi: 10.1109/LPT.2006.871110

[9] S. I. Yakovlenko: "Mechanism for the void formation in the bright spot of a fiber fuse", Laser Physics, **16**, 3, pp. 474–476 (2006). doi: 10.1134/S1054660X0603008X Homepage

[10] Y. Shuto, S. Yanagi, S. Asakawa, M. Kobayashi and R. Nagase: "Fiber fuse phenomenon in triangular-profile single-mode optical fibers", J. Lightwave Technol., **24**, 2, pp. 846–852 (2006). Download

[11] S. I. Yakovlenko: "About the mechanism of formation of a chain of caverns in plasma behind a bright spot of the fiber fuse", Proceedings of Annual Moscow Workshop on the Non-ideal Plasma Physics, Moscow, Russia (2005). (P-7). Homepage

[12] E. D. Bumarin and S. I. Yakovlenko: "The temperature distribution in a bright spot of the fiber fuse", Proceedings of Annual Moscow Workshop on the Non-ideal Plasma Physics, Moscow, Russia (2005). (P-8). Homepage

[13] S. Todoroki: "Transient propagation mode of fiber fuse leaving no voids", Optics Express, **13**, 23, pp. 9248–9256 (2005). doi: 10.1364/OPEX.13.009248

[14] S. Todoroki: "Animation of fiber fuse damage, demonstrating periodic void formation", Opt. Lett., **30**, 19, pp. 2551–2553 (2005). doi: 10.1364/OL.30.002551 PDFfile

[15] I. A. Bufetov, A. A. Frolov, V. P. Efremov, M. Ya. Schelev, V. I. Lozovoi, V. E. Fortov and E. M. Dianov: "Fast optical discharge propagation through optical fibres under kW-range laser radiation", Proceedings of the 31st European Conference on Optical Communication, Vol. 6, Glasgow, Scotland, IEE's Photonics Professional Network, pp. 39–40 (2005). (Th 4.4.2). Homepage

[16] E. M. Dianov, I. A. Bufetov, A. E. Rakitin, M. A. Melkumov, A. A. Frolov, V. E. Fortov, V. P. Efremov and M. I. Kulish: "Temperature of optical discharge under action of laser radiation in silica-based fibres", Proceedings of the 31st European Conference on Optical Communication, Vol. 3, Glasgow, Scotland, IEE's Photonics Professional Network, pp. 469–470 (2005). (We 3.4.4). Homepage

[17] S. Todoroki: "Origin of periodic void formation during fiber fuse", Optics Express, **13**, 17, pp. 6381–6389 (2005). doi: 10.1364/OPEX.13.006381

[18] R. I. Golyatina and S. I. Yakovlenko: "On the mechanism of optical discharge stop in the tapered region of fibre cladding", Quantum Electron., **35**, 5, pp. 422–424 (2005). doi: 10.1070/QE2005v035n05ABEH003425

[19] E. M. Dianov, V. E. Fortov, I. A. Bufetov, V. P. Efremov, A. E. Rakitin, M. M. Melkumov, M. I. Kulish and A. A. Frolov: "Temperature of plasma in silica-based fibers under the action of CW laser radiation", International Conference on Strongly Coupled Coulomb Systems Book of Abstracts, Moscow, Russia, pp. 25–26 (2005). (P23). Homepage

[20] S. Todoroki: "In-situ observation of fiber-fuse propagation", Jpn. J. Appl. Phys., **44**, 6A, pp. 4022–4024 (2005). doi: 10.1143/JJAP.44.4022

2 Fiber Fuse (ICONO/LAT2005)

[1] S. Todoroki: "Ultrahigh-speed videography of fiber fuse propagation: a tool for studying void formation", International Conference on Lasers, Applications, and Technologies 2005: Laser-Assisted Micro- and Nanotechnologies, Vol. 6161 of SPIE Proceedings, SPIE, pp. 61610L–1–8 (2006). (St. Petersburg, Russia, 15 May 2005, LSuH1). Download BookStore Homepage

[2] S. I. Yakovlenko: "Plasma in bright spot and nature of void chain in fiber fuse track", International Conference on Lasers, Applications, and Technologies 2005: Laser-Assisted Micro- and Nanotechnologies (Eds. by V. I. Konov, V. Y. Panchenko, K. Sugioka and V. P. Veiko), Vol. 6161 of SPIE Proceedings, SPIE, pp. 61610M–1–6 (2006). (St. Petersburg, Russia, 15 May 2005, LSuH2, in print). Download BookStore Homepage

[3] I. A. Bufetov, E. M. Dianov, A. A. Frolov, V. E. Fortov and V. P. Efremov: "Dynamics of optical discharge propagation along a fiber", ICONO/LAT 2005 Technical Digest on CD-ROM, St. Petersburg, Russia (2005). (LSuH3). Homepage

[4] A. A. Frolov, I. A. Bufetov and E. M. Dianov: "Propagation of the optical discharge through the fibers with thin silica cladding", ICONO/LAT 2005 Technical Digest on CD-ROM, St. Petersburg, Russia (2005). (LSuH4). Homepage

[5] R. I. Golyatina, A. N. Tkachev and S. I. Yakovlenko: "2D calculation of a fiber fuse propagation", ICONO/LAT 2005 Technical Digest on CD-ROM, St. Petersburg, Russia (2005). (LSuH5). Homepage

[6] S. Todoroki: "In-situ observation of fiber-fuse ignition", International Conference on Lasers, Applications, and Technologies 2005: Laser-Assisted Micro- and Nanotechnologies (Eds. by V. I. Konov, V. Y. Panchenko, K. Sugioka and V. P. Veiko), Vol. 6161 of SPIE Proceedings, SPIE, pp. 61610N–1–4 (2006). (St. Petersburg, Russia, 14 May 2005, LSK3). Download BookStore Homepage

[7] S. I. Yakovlenko: "Plasma behind a front of a wave of optical fiber destruction and the nature of occurrence of periodic emptiness under influence of laser radiation", ICONO/LAT 2005 Technical Digest on CD-ROM, St. Petersburg, Russia (2005). (IFM4). Homepage

[8] S. I. Yakovlenko: "About the reasons of strong photoabsorption in an optical fiber at the high temperature", ICONO/LAT 2005 Technical Digest on CD-ROM, St. Petersburg, Russia (2005). (IFM5). Homepage

[9] R. I. Golyatina, A. N. Tkachev and S. I. Yakovlenko: "2D calculation of an absorption thermal wave of laser radiation in an optical fiber", ICONO/LAT 2005 Technical Digest on CD-ROM, St. Petersburg, Russia (2005). (IFM6). Homepage

[10] A. A. Frolov, E. M. Dianov and I. A. Bufetov: "Destruction of silica fiber cladding by the optical discharge propagation", ICONO/LAT 2005 Technical Digest on CD-ROM, St. Petersburg, Russia (2005). (LThI6). Homepage

[11] I. A. Bufetov, E. M. Dianov and A. A. Frolov: "Optic discharge propagation along a fiber core", ICONO/LAT 2005 Technical Digest on CD-ROM, St. Petersburg, Russia (2005). (LWG4). Homepage

3 Fiber Fuse (2001-2005)

[1] I. A. Bufetov, A. A. Frolov, E. M. Dianov, V. E. Fortov and V. P. Efremov: "Dynamics of fiber fuse propagation", Optical Fiber Communication Conference, 2005. Technical Digest. OFC/NFOEC, Vol. 4, Anaheim, CA (2005). (OThQ7). Download Homepage

[2] I. A. Bufetov and E. M. Dianov: "Optical discharge in optical fibers", Physics-Uspekhi, **48**, 1, pp. 91–94 (2005). doi: 10.1070/PU2005v048n01ABEH002081 Homepage

[3] I. A. Bufetov and E. M. Dianov: "Optical discharge in optical fibers", Lightwave Russian Edition, **29**, 4, pp. 50–51 (2004). (in Russian). Download Homepage

[4] S. Todoroki: "In-situ observation of fiber-fuse propagation", Proc. 30th European Conf. Optical Communication Post-deadline papers, Stockholm, Sweden, Kista Photonics Research Center, pp. 32–33 (2004). (Th4.3.3). Homepage

[5] S. I. Yakovlenko: "On reasons for strong absorption of light in an optical fibre at high temperature", Quantum Electron., **34**, 9, pp. 787–789 (2004).
doi: 10.1070/QE2004v034n09ABEH002762

[6] R. I. Golyatina, A. N. Tkachev and S. I. Yakovlenko: "Analysis of a heat wave induced by laser radiation absorption in an optical fiber on the basis of a 2D nonstationary heat conduction equation", Tech. Phys., **50**, 2, pp. 232–236 (2005). (Zh. Tech. Fiz. 75 (2) 94 (2005)).
Homepage

[7] R. I. Golyatina, A. N. Tkachev and S. I. Yakovlenko: "Calculation of velocity and threshold for a thermal wave of laser radiation absorption in a fiber optic waveguide based on the two-dimensional nonstationary heat conduction equation", Laser Physics, **14**, 11, pp. 1429–1433 (2004).
Homepage

[8] E. M. Dianov, I. A. Bufetov and A. A. Frolov: "Destruction of silica fiber cladding by the fuse effect", Opt. Lett., **29**, 16, pp. 1852–1854 (2004). doi: 10.1364/OL.29.001852

[9] E. M. Dianov, I. A. Bufetov and A. A. Frolov: "Destruction of silica fiber cladding by the fuse effect", OFC 2004 Technical Digest, Los Angels (2004). (TuB4). Homepage

[10] S. I. Yakovlenko: "Plasma behind the front of a damage wave and the mechanism of laser-induced production of a chain of caverns in an optical fibre", Quantum Electron., **34**, 8, pp. 765–770 (2004). doi: 10.1070/QE2004v034n08ABEH002845

[11] A. N. Tkachev and S. I. Yakovlenko: "Calculation of the velocity and threshold of a thermal absorption wave of laser radiation in an optical fibre", Quantum Electron., **34**, 8, pp. 761–764 (2004). doi: 10.1070/QE2004v034n08ABEH002844

[12] Y. Shuto, S. Yanagi, S. Asakawa, M. Kobayashi and R. Nagase: "Fiber fuse phenomenon in step-index single-mode optical fibers", IEEE J. Quantum Electronics, **40**, 8, pp. 1113–1121 (2004). doi: 10.1109/JQE.2004.831635

[13] Y. Shuto, S. Yanagi, S. Asakawa, M. Kobayashi and R. Nagase: "Evaluation of high-temperture absorption coefficients of optical fibers", IEEE Photon. Technol. Lett., **16**, 4, pp. 1008–1010 (2004). doi: 10.1109/LPT.2004.824633

[14] N. Nishimura, K. Seo, M. Shiino and T. Shiba: "Study of high-power endurance characteristics in optical fiber link", Optical Amplifiers and Their Applications, 2003, Trends in Optics and Photonics, Vol. 92, Optical Society of America, pp. 94–100 (2003).

[15] N. Nishimura, K. Seo, M. Shiino and R. Yuguchi: "Study of high-power endurance characteristics in optical fiber link", Technical Digest of Optical Amplifiers and Their Applications, pp. 193–195 (2003). (TuC4). Homepage

[16] S. Yanagi, S. Asakawa, M. Kobayashi, Y. Shuto and R. Naruse: "Fiber fuse terminator", The 5th Pacific Rim Conference on Lasers and Electro-Optics, Vol. 1, p. 386 (2003). (W4J-(8)-6, Taipei. Taiwan, 22-26 Jul. 2003). Download

[17] Y. Shuto, S. Yanagi, S. Asakawa, M. Kobayashi and R. Nagase: "Fiber fuse generation in single-mode fiber-optic connectors", IEEE Photon. Techol. Lett., **16**, 1, pp. 174–176 (2004).
doi: 10.1109/LPT.2003.820479

[18] E. M. Dianov, A. A. Frolov, I. A. Bufetov, S. L. Semenov, Y. K. Chamorovskii, G. A. Ivanov and I. L. Vorob'ev: "The fibre fuse effect in microstructured fibres", Quantum Electron., **34**, 1, pp. 59–61 (2004). doi: 10.1070/QE2004v034n01ABEH002581

[19] K. Seo, N. Nishimura, M. Shiino, R. Yuguchi and H. Sasaki: "Evaluation of high-power endurance in optical fiber links", Furukawa Review, 24, pp. 17–22 (2003). Download Homepage

[20] K. Seo, N. Nishimura, M. Shiino, R. Yuguchi and H. Sasaki: "Examination of threshold power for high-power problems in optical fiber", Proc. Int. Laser Safety Conf., pp. 298–302 (2003).

[21] Y. Shuto, S. Yanagi, S. Asakawa, M. Kobayashi and R. Nagase: "Simulation of fiber fuse phenomenon in single-mode optical fibers", J. Lightwave Tech., **21**, 11, pp. 2511–2517 (2003). doi: 10.1109/JLT.2003.819142

[22] R. Kashyap: "High average power effects in optical fibers and devices", Reliability of Optical Fiber Components, Devices, Systems, and Networks (Eds. by H. G. Limberger and M. J. Matthewson), Vol. 4940 of SPIE Proceedings, SPIE, pp. 108–117 (2003). (Brugge, Belgium, 28 Oct. 2002). doi: 10.1117/12.477395

[23] R. M. Atkins, P. G. Simpkins and A. D. Yablon: "Track of a fiber fuse: a rayleigh instability in optical waveguides", Opt. Lett., **28**, 12, pp. 974–976 (2003). doi: 10.1364/OL.28.000974

[24] Y. Shuto, S. Yanagi, S. Asakawa and R. Nagase: "Generation mechanism on fiber fuse phenomenon in single-mode optical fibers", The Institute of Electronics, Information and Communication Engineers, **J86-C**, 3, pp. 252–261 (2003). (In Japanese). Homepage

[25] E. M. Dianov, I. A. Bufetov, A. A. Frolov, Y. K. Chamorovsky, G. A. Ivanov and I. L. Vorobjev: "Fiber fuse effect in microstructured fibers", IEEE Photon. Technol. Lett., **16**, 1, pp. 180–181 (2004). doi: 10.1109/LPT.2003.820465

[26] E. Dianov, A. Frolov, I. Bufetov, Y. Chamorovsky, G. Ivanov and I. Vorobjev: "Fiber fuse effect in microstructured fibers", OFC 2003 Technical Digest, Vol. 2, Atlanta (2003). (FH2). Download

[27] S. Yanagi, S. Asakawa and R. Naruse: "Characteristics of fiber-optic connector at high-power optical incidence", Electron. Lett., **38**, 17, pp. 977–978 (2002). doi: 10.1049/el:20020647

[28] E. M. Dianov, I. A. Bufetov, A. A. Frolov, V. G. Plotnichenko, V. M. Mashinskii, M. F. Churbanov and G. E. Snopatin: "Catastrophic destruction of optical fibres of various composition caused by laser radiation", Quantum Electron., **32**, 6, pp. 476–478 (2002). doi: 10.1070/QE2002v032n06ABEH002226

4 Fiber Fuse (-2000)

[1] R. M. Percival, E. S. R. Sikora and R. Wyatt: "Catastrophic damage and accelerated ageing in bent fibres caused by high optical powers", Electron. Lett., **36**, 5, pp. 414–416 (2000). doi: 10.1049/el:20000403

[2] D. D. Davis, S. C. Mettler and D. J. DiGiovani: "A comparative evaluation of fiber fuse models", Laser-Induced Damage in Optical Materials: 1996 (Eds. by H. E. Bennett, A. H. Guenther, M. R. Kozlowski, B. E. Newnam and M. J. Soileau), Vol. 2966 of SPIE Proceedings, SPIE, pp. 592–606 (1997). (Boulder, CO, USA, 7 Oct 1996). doi: 10.1117/12.274220

[3] R. Kashyap, A. Sayles and G. F. Cornwell: "Heat flow modeling and visualization of catastrophic selfpropagating damage in singlemode optical fibers at low powers", Laser-Induced Damage in Optical Materials: 1996 (Eds. by H. E. Bennett, A. H. Guenther, M. R. Kozlowski, B. E. Newnam and M. J. Soileau), Vol. 2966 of SPIE Proceedings, SPIE, pp. 586–591 (1997). (Boulder, CO, USA, 7 Oct 1996). doi: 10.1117/12.274219

[4] D. D. Davis, S. C. Mettler and D. J. DiGiovani: "Experimental data on the fiber fuse", 27th Annual Boulder Damage Symposium: Laser-Induced Damage in Optical Materials: 1995 (Eds. by H. E. Bennett, A. H. Guenther, M. R. Kozlowski, B. E. Newnam and M. J. Soileau), Vol. 2714 of SPIE Proceedings, SPIE, pp. 202–210 (1996). (Boulder, CO, USA, 30 Oct. 1995). doi: 10.1117/12.240382

[5] E. M. Dianov, V. M. Mashinskii, V. A. Myzina, Y. S. Sidorin, A. M. Streltsov and A. V. Chickolini: "Change of refractive index profile in the process of laser-induced fiber damage", Sov. Lightwave Commun., **2**, pp. 293–299 (1992).

[6] T. J. Driscoll, J. M. Calo and N. M. Lawandy: "Explaining the optical fuse", Opt. Lett., **16**, 13, pp. 1046–1048 (1991). Download

[7] D. P. Hand and T. A. Birks: "Single-mode tapers as 'fibre fuse' damage circuit-breakers", Electron. Lett., **25**, 1, pp. 33–34 (1989). Download

[8] D. P. Hand and P. St. J. Russell: "Soliton-like thermal shock-waves in optical fibers: origin of periodic damage tracks", Eur. Conf. Optical Communications, pp. 111–114 (1988). (Brighton, UK, 11-15 Sep 1988). Download

[9] D. P. Hand and P. St. J. Russell: "Solitary thermal shock waves and optical damage in optical fibers: the fiber fuse", Opt. Lett., **13**, 9, pp. 767–769 (1988). Download

[10] R. Kashyap and K. J. Blow: "Observation of catastrophic self-propelled self-focusing in optical fibres", Electron. Lett., **24**, pp. 47–49 (1988). doi: 10.1049/el:19880032

[11] R. Kashyap: "Self-propelled self-focusing damage in optical fibres", Lasers '87; Proc. the Tenth Int. Conf. Lasers and Applications, McLean, VA, STS Press, pp. 859–866 (1988). (Lake Tahoe, NV, Dec. 7-11, 1987). Homepage

[12] R. Kashyap and K. J. Blow: "Spectacular demonstration of catastrophic failure in long lengths of optical fibre via self-propelled self-focusing", Eighth National Quantum Electronics Conference, Univ. of St Andrews, p. PD7 (1987). (21–25 Sept.).
