# Supplementary material for: In Situ Observation of Modulated Light Emission of Fiber Fuse Synchronized with Void Train over Hetero-Core Splice Point
Source: PLoS One. 2008 Sep 25;3(9):e3276. doi: 10.1371/journal.pone.0003276 (PMC2542984; doi:10.1371/journal.pone.0003276)
Supplement: Text S2 — Japanese translation of this paper. (1.11 MB PDF) [file pone.0003276.s002.pdf]

異径コア融着点を通過するファイバヒューズのその場観察と空孔列の変調パターンとの関係

轟 眞市
物質・材料研究機構 光材料センター
茨城県つくば市並木 1-1
TODOROKI.Shin-ichi at nims.go.jp

http://www.geocities.jp/tokyo_1406/

要旨: 異径コア (Corning SMF-28e と HI 1060) 融着点付近を通過するファイバヒューズをその場観察した。1070 nm 9 W の光を供給した光学的放電を 2.78 μs 毎に記録した一連の写真から、その末尾に周期的発光が現れることがわかった。この信号は、異径コア融着点を横切る時には現れない。この時にコア領域に残される損傷パターンには、周期性が欠落した区間が認められた。いくつかの区間には、レーザー光の進行方向とは逆の方向に向いた弾丸状空孔が現れたが、ビデオ映像では光学的放電が進行方向を変えた形跡は残されていなかった。

2008 年 7 月 4 日提出、2008 年 9 月 4 日採択、2008 年 9 月 25 日発行

本論文は、PLoS ONE に投稿した原稿 “In situ observation of modulated light emission of fiber fuse synchronized with void train over hetero-core splice point”, の和訳である。http://dx.plos.org/10.1371/journal.pone.0003276

参考文献とリンク

1. R. Kashyap and K. J. Blow, “Observation of catastrophic self-propelled self-focusing in optical fibres,” Electron. Lett. **24**, 47–49 (1988).
2. D. P. Hand and P. St. J. Russell, “Solitary thermal shock waves and optical damage in optical fibers: the fiber fuse,” Opt. Lett. **13**(9), 767–769 (1988).
3. S. Todoroki, “Animation of fiber fuse damage, demonstrating periodic void formation,” Opt. Lett. **30**(19), 2551–2553 (2005).
4. S. Todoroki, “Origin of periodic void formation during fiber fuse,” Optics Express **13**(17), 6381–6389 (2005).
5. S. Todoroki, “Transient propagation mode of fiber fuse leaving no voids,” Optics Express **13**(23), 9248–9256 (2005).
6. I. A. Bufetov, A. A. Frolov, A. V. Shubin, M. E. Likhachev, C. V. Lavrishchev, and E. M. Dianov, “Fiber fuse effect: New results on the fiber damage structure,” in *Proceedings of the 33rd European Conference on Optical Communication*, vol. 1, pp. 79–80 (IEE’s Photonics Professional Network, Berlin, Germany, 2007). (Mon 1.5.2).

---

1. 緒言

ファイバヒューズ効果は 1980 年代末に最初の報告がなされている [1, 2]. 数 W の光を伝搬している光ファイバの局所的加熱によって発生し、生成した光学的放電は光源に向かって走り始める。これはコア領域の壊滅的な破壊をもたらす。よってこの現象は、強力光と

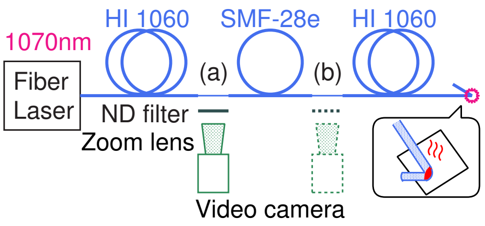

Fig. 1. 異径コア融着点前後のファイバヒューズ伝搬の観察に用いた実験設備。

光導波路を用いるすべてのシステムにとって真の脅威となっている。関係する 70 報の一覧は筆者のホームページで公開している。

高速に移動する光学的放電の振る舞いを知ることは、損傷の生成を理解する鍵となる。よって、毎秒 50 万フレーム程度の超高速ビデオ撮影はこの振る舞いを観察するのに最適な道具である。筆者はこの技術を用いて光学的放電を撮影し、また冷却後の空孔の顕微鏡写真と組み合わせて、単一モードシリカガラスファイバに 1480 nm の光を供給した時の空孔生成メカニズムを決定した [3, 4, 5]。

この仕事においてビデオ撮影から得た情報は、光学的放電の動きは 4 μ 間隔で見る限り等速運動している、という点だけであった。本論文では、最新式のカメラを用いて光学的放電からの発光の変動を捉え、それが異径コア融着点を通過するときに残る不規則な空孔列と関係することを示す。さらに、通常の弾丸型空孔の向きとは逆の方向に向いた空孔が融着点付近に偶然見付かったが、光学的放電の移動方向の変化は認められなかった。これらの情報は、高出力ファイバレーザーのファイバーヒューズ発生事故の解析に有用である。

2.　実験

Figure 1 に、異径コア融着点を通過するファイバヒューズを観察するための実験設備を示す。商用の単一モードシリカガラスファイバ (SMF-28, Corning, Table 1 参照) の一端をラマンファイバレーザ (PLM-10-1070, IPG Laser, 1.07 μm, 9 W) の出力ポートとなる光ファイバ (HI 1060, Corning) に接続した (Table 1 参照)。もう一方の光ファイバの端を、別の HI 1060 ファイバに融着した。以下、これら 2 つの接続点を、それぞれ (a)、(b) と記す。異径コア融着は、専用プログラムが内蔵された融着接続器 (S183, Furukawa Electric) で行った。

表 1. 本研究で用いたファイバーの仕様。

	HI 1060	SMF-28e
カットオフ波長	920±50 nm	$\leq$ 1280 nm
モードフィールド径	6.2±0.3 μm @ 1060 nm	9.2±0.4 μm @ 1310 nm
コア径	n. a.	8.2 μm
クラッド径	125 μm	125 μm

出典: 製品情報シート http://www.corning.com/opticalfiber/

ファイバヒューズはファイバ端を金属板に接触させることで発生させた。2 つある融着点の一方を通過する様子を超高速 CCD カメラ (FASTCAM SA1.1, モノクロ白黒バージョ

ン, Photron Ltd., 感度域: 380-790 nm) に適当な倍率のズームレンズを取り付けて観察した。画像は 1024 階調の 256×32 ドットの分解能に設定し、8 倍の ND フィルタを通して露光時間を 0.37μ 秒とし、2.78 μ 秒毎 (360,000 frames per second) に撮影した。 損傷領域は光学顕微鏡で観察した。

3. 結果

ファイバヒューズ伝搬を十例記録したが、どれも同じ傾向を示した。典型的な記録を Fig. 2 (a) と (b) に示す。図の上段は、各融着点上を通過する光学的放電から放射される可視光である。ファイバか円柱状レンズとして働くので、これらの映像は縦方向に拡大されている。図の中段は、その上の図内の点線上の発光強度分布の時間変化を示している。

下段の写真は伝搬後に残された損傷列である。融着点は白丸の付近に位置していると思われる。空孔列を見れば、光学的放電の伝搬モードは融着点付近で変調されていることがわかる。これに伴い、その速度は 2 度変化した。すなわち、融着点に到達する前と後である。速度が変化する場所をビデオ映像の解析から特定し、中段の図中の縦の点線で示した。この線の位置は、速度変化を起こす位置における強度分布のピークに相当する。

それぞれの区間における平均速度 v を表 2 に示す。下流側の HI 1060 における速度が、上流側のそれよりも小さくなっているのは、2 つの異径コア融着点における避けられない挿入損失によって、供給エネルギーが減衰するためである。

表 2. 図 2 に示した融着点付近における、光学的放電の平均速度, v, および空孔 1 個の生成に要する平均時間, τ。

	Fig. 2 (a)				Fig. 2 (b)		
	HI 1060	···	SMF-28e	∼	SMF-28e	···	HI 1060
v (m/s)	2.08	1.69	1.42		1.40	1.51	1.88
τ (μs)	6.6	—	12.1		16.7	—	8.4

中段に示した強度分布の末尾には、小さな独立したピークが周期的に現れ、高さを減じながら後方に移動する様が観察された。この挙動は融着点付近では見られなかった。このサブピークの振動は筆者の前の仕事 [4] でも確認されたが、ビデオカメラの分解能が粗かったために (256 階調、4 μ 秒間隔)、詳細な分析は行わなかった。このピークの振動周期は、表 2 に示した、空孔 1 個の生成に要する時間, τ, と対応している。なお、τ は平均速度と空孔間隔から算出した。

時々、単一の逆向き弾丸が融着点 (a) の手前で現れることがあった (Fig. 2(a-3) 参照). この拡大像を、同じ条件で得た他の試料写真と共に Fig. 3 に示す。

4. 考察

4.1. 融着点前後の空孔パターンの変化

供給光 (1070 nm) は SMF-28e 中をマルチモードで伝搬するため、そのエネルギー密度は HI 1060 中のものより低い。よって、光学的放電の伝搬速度は Table 2 に示す様に SMF-28e の方が小さい。融着点の前後で 2 度速度が変化する理由は、光学的放電は 100μm 以上の

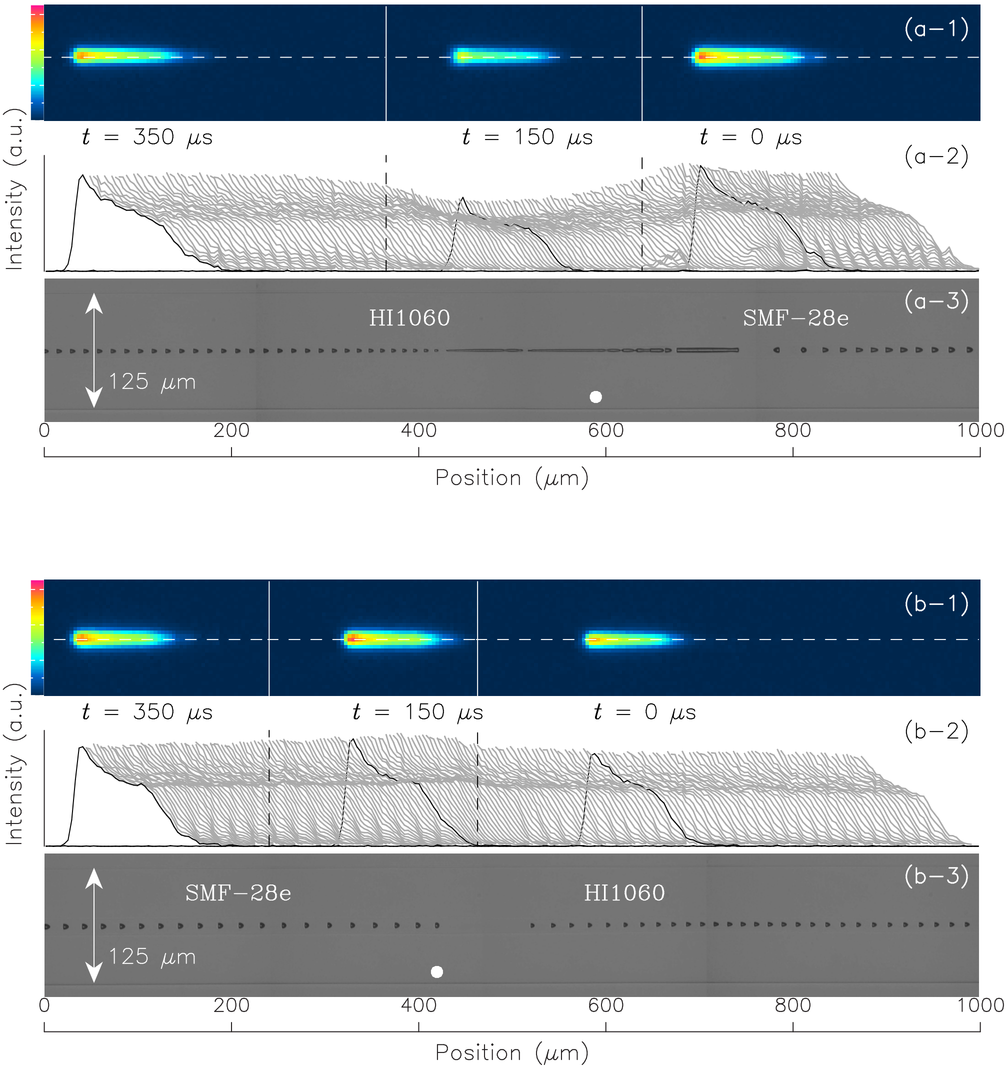

Fig. 2. 実験結果のまとめ ((a) 348 kB and (b) 390 kB)。 1070 nm 9W の光を供給したファイバヒューズが 2 つの融着点 (a) および (b) を通過する時に発する可視光の写真 (上, 元のグレースケール画像をカラースケール画像に変換してある。), その写真中の点線に沿った強度分布を 2.78 μ 秒間隔で並べたもの (中)、および屈折液に浸して撮影した損傷パターンの光学顕微鏡写真。各融着点は白丸の付近に位置している。クラッド径は 125 μm。2 本の縦点線は、光学的放電の移動速度が変化した場所を示す (本文参照)。

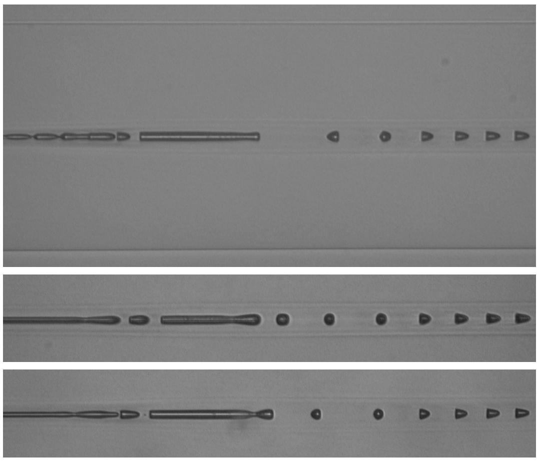

Fig. 3. 融着点 (a) 付近に生成されたファイバーヒューズ損傷の光学顕微鏡写真。供給レーザー光は 1070 nm、9.0 W。上の写真は Fig. 2 (a) の拡大画像。

長さがあるので、境界線上を走る時には HI 1060 と SMF-28e の両方に滞在する過渡的状態になるからである。

この過渡的区間 (Fig.2 の 2 本の縦点線で示された区間) では、融着点 (Fig.2 の白丸) は、供給レーザーに対して下流側に位置している。これは、光学的放電のファイバ長に沿った強度分布が非対称なためである。

過渡的区間の外側では、光学的放電は周期的な空孔列を生成する。融着点 (b) の通過によって、光学的放電は空孔生成頻度が小さくなる (Table 2 参照)。しかし、その境界付近では周期的空孔の間隔は不連続に変化し、無空孔区間が生じる。

ファイバヒューズ伝搬時のエネルギー収支を考えると、入力は供給レーザーのエネルギーであり、出力は物質の加熱、光と熱の放射、光学的放電の移動、そして空孔表面の生成である。よって、過渡的区間では、別の平衡状態を確立するためにこれらのバランスが変化する。境界 (b) を通過する間に、光学的放電は空孔生成を止め、光放射をわづかに増加させる。同様の現象はファイバヒューズの自然停止においても観察されている [5]。

他方、境界 (a) を通過すると光学的放電は周期的空孔の生成頻度を増やす。よって、過渡的区間の放電は表面をより多く生成して長い空孔列を作りつつ、光放射を減じている。

両方の過渡的区間において、光学的放電は、その末尾に現れるサブピークの周期的振動も停止させている。これは、Fig. 2 (a-2) および (b-2) に示した強度分布の重ね合わせによる模様にはっきり現れている。ピークの振動は縞模様で現れているが、これら過渡的区間では消えている。 この縞模様の間隔は、Fig. 2 (a-3) および (b-3) に示した空孔列の間隔と一致している。実際のところ、ビデオ映像からは融着点の情報は得られないが、これらの縞模様のおかげで空孔写真と正確に重ね合わせることができる。

以上により、サブピークの振動は周期的空孔生成と関係しているのは明らかである。

4.2.　損傷列中の弾丸形状の方向

通常のファイバ中に生成される弾丸状の空孔は、供給レーザー光の伝搬方向と同じ方向に向くことは良く知られている。この形状の形成メカニズムは、光学的放電の内圧と、ファイバに沿った温度勾配が結び付いた効果で説明できる [3]。一つの空孔が光学的放電の末尾から切り離されると、低温側の表面は球面を保って先に固まるのに対し、反対の高温側は圧を受けて平面を形成するのである。

しかし Bufetov らの報告によると、ファイバコアに沿った強度分布 (LP_{01} と LP_{02} モードの干渉) が存在する時に逆向きの空孔生成するという [6]。本研究では、Fig. 3 に示すように、逆向きの空孔が融着点 (a) の近くで時々観察された。ビデオ映像によると、光学的放電は方向転換を起こしていないので、この形成メカニズムは上述したものとは異なっていると言える。実際、Fig. 2 (a-2) の逆向き空孔付近の縞模様は、他の周期的空孔の位置に現れるものと異なっている。よって、この現象も、異径コア融着によって光強度が変調されたことが原因で起こると考えられる。

5.　結論

異径コア融着点を通過する光学的放電の末尾に現れる発光の振動をその場観察した。その空間的変調パターンは、異径コア融着点を跨いで形成される空孔列と一致した。融着点のそばで逆向きの弾丸状空孔が時々生成される場合があった。この領域での、ファイバヒューズの進行方向転換は検出されなかった。

謝辞

超高速ビデオ撮影にご協力頂いた、株式会社フォトロンの相澤啓助氏、近藤圭紘氏、花香和秀氏に謝意を表します。
